# Intravenous Lipid Emulsion for Managing Local Anesthetic Toxicity in a Three-Month-Old Patient

**DOI:** 10.7759/cureus.75981

**Published:** 2024-12-18

**Authors:** Abdulrahman S Alquzi, Bader Alsabban, Ahmed Bokhari

**Affiliations:** 1 Anesthesia Department, King Abdulaziz Medical City, Jeddah, SAU

**Keywords:** lipid emulsion, local anesthetic systemic toxicity (last), pediatric case, pediatric regional anesthesia, regional anesthesia

## Abstract

Local anesthetic systemic toxicity (LAST) is a well-known life-threatening local anesthetics complication, especially if given in inappropriate doses or routes. Therefore, physicians should be aware of LAST symptoms, such as neurological and cardiac symptoms. In addition, they should always consider it in the differential diagnosis when they encounter similar symptoms. The early recognition of LAST and initiation of intravenous lipid emulsion (ILE) can decrease mortality and morbidity rates. Multiple organizations and guidelines use ILE as standard LAST management.

We present a three-month-old infant who received a caudal block that was complicated by an inadvertent intravascular injection that led to LAST cardiac symptoms. ILE successfully managed these symptoms, and the patient returned to normal vital signs without any complications.

## Introduction

Local anesthetics have become a major part of the anesthesia practice due to their varied use for local wound infiltration, neuraxial blocks, and regional blocks. Despite the good safety profile for local anesthetics, their use can lead to significant morbidity and mortality. Local anesthetic systemic toxicity (LAST) is one of the most common complications of local anesthetics due to inadvertent intravascular injection or overdose. LAST affects cardiovascular and neurological systems, with fatal outcomes if not recognized and treated properly. Since its first use in a case for LAST in 2006, intravenous lipid emulsion (ILE) has become the standard treatment for LAST according to multiple recommendations from different anesthesia societies. Since there are a few case reports about ILE usage and effectiveness in reversing the cardiotoxic effects of bupivacaine in infants, this report aims to contribute to the existing literature by presenting a case of a three-month-old male infant who developed LAST following a caudal block for a circumcision procedure and was successfully treated with ILE.

## Case presentation

The patient, a 5.8-kg, three-month-old infant with an insignificant medical or surgical history, was admitted as a day surgery case for a circumcision procedure. The patient underwent induction of general anesthesia with propofol and rocuronium and was intubated uneventfully.

**Figure 1:**
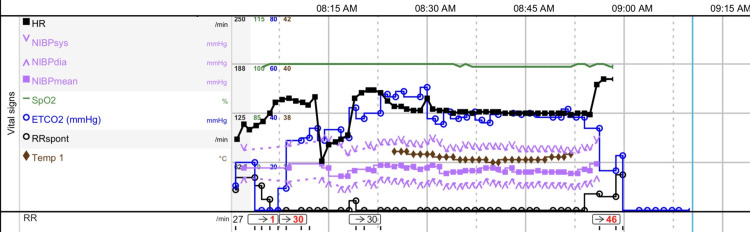
Vital signs of the patient during the procedure. Please note the drop in heart rate after the caudal block.

After induction, the patient was prepared for caudal anesthesia for postoperative pain management. He was positioned on left lateral decubitus, and 8 mL of 0.25% bupivacaine mixed with adrenaline (5 mcg/mL) was to be given. After negative aspiration, 3 mL was given slowly, followed by a second negative aspiration and an additional 3 mL. However, with the final aspiration, blood was noted (6 mL administered, 2 mL remaining), and the caudal procedure was aborted immediately. This was followed by a sudden drop in heart rate from 130 to 65 bpm with a bigeminy rhythm; blood pressure remained adequate, and bupivacaine toxicity was suspected (Figure 1).

Help was immediately called for, and intralipid 20% was requested. During this time, heart rate increased to 130-140 bpm but still with bigeminy. Within 15 minutes, 9 mL of intralipid 20% was administered, the rhythm slowly returned to normal sinus rhythm, and the intralipid 20% infusion was started. Patient's condition was deemed stable and the surgery was completed uneventfully. The patient was extubated and was breathing spontaneously.

The anesthetist recommended admitting the patient for observation. During this time, the patient’s vitals were stable, no arrhythmia or seizure was reported, and investigations were all within normal limits, including complete blood count, electrolytes, CRP, and kidney function tests. The patient was discharged after two days with a follow-up appointment in the pediatric clinic.

## Discussion

Using ILE to treat cardiac symptoms from bupivacaine toxicity has shown to be effective in multiple case reports and systematic reviews in most adult cases [1]. In pediatrics, so far, there are 21 cases reported using ILE to reverse LAST symptoms. Among those 21 reported cases, only nine were infants below one year of age [2]. In this study, the patient received an inadvertent intravascular injection of bupivacaine 6 mL of 0.25% mixed with adrenaline 5 mcg/mL. Despite the negative aspiration test in the first injections, the patient had immediate bradycardia and arrhythmia, specifically diagnosed as bigeminy. There were no noticeable neurological symptoms because the patient was under general anesthesia when performing caudal block. After giving 9 mL of intralipid 20% as a bolus over three minutes followed by infusion, the heart rate slowly improved and returned to sinus rhythm and full recovery. This result supports ILE’s effectiveness at prompting a rapid recovery from LAST [2].

Among the local anesthetics that cause LAST and cardiac depression, bupivacaine is considered the most cardiotoxic [3]. In addition, bupivacaine was reported as the most common medication to cause LAST in pediatric patients [4]. The injection site and its associated vascularity can contribute to LAST’s development. As in this case, caudal block was the reported injection site in 29% of cases in children who experienced LAST [2]. Ultrasound (US) use is recommended while performing caudal block as the negative aspiration test with or without adrenaline is reported to be considerably inaccurate in pediatrics [4]. In the present case, unfortunately, the US was not used. LAST is frequently found in infants less than one year of age who receive ILE therapy [5]. The amide class of local anesthetics, such as bupivacaine and ropivacaine, are highly bound to alpha-1-acid glycoprotein (AAG) and loosely bound to albumin [6]. Infants less than one year of age have 20-50% of an adult’s level of AAG, and that leads to high levels of unbound amide local anesthetics. In addition to low AAG levels, they have immature hepatic enzymes [7-9]. The potential toxicity of local anesthetics is higher in infants due to their decreased clearance and increased terminal half-life, which is three to eight times longer compared to adults, owing to lower AAG levels and immature hepatic enzymes [7-9]. The accepted theory for ILE’s mechanism of action is the lipid shuttle. ILE creates a lipid compartment that reduces the level of lipid-soluble local anesthetics, such as bupivacaine, that are sent to the brain, heart, and other body organs [10]. After ILE absorbs them, the local anesthetics are moved to the liver, adipose tissues, and muscles where they get metabolized [10].

## Conclusions

This case illustrates the significance of clinical attentiveness and draws attention to a rare but dangerous side effect of using local anesthetics. Moreover, in order to reduce the risk of potentially fatal consequences, this case serves as a reminder of the importance of following the recommendation of using the aspiration technique every 3 mL of injection, guided by ultrasound and careful monitoring both before and after the administration of intralipid or local anesthetic, particularly in pediatric patients. Anesthesiologists and other medical professionals should maintain a low threshold for suspicion of LAST and recognize its symptoms, such as altered mental status, seizures, or cardiac abnormalities, keeping in mind that the presentation may be different and more subtle in pediatric patients compared to the adult population. The occurrence of these symptoms straight after administration of local anesthetics further increases the suspicion of LAST. Early initiation of ILE can successfully manage LAST and prevent further complications.

To enhance preparedness, hospitals should keep ILE readily available, such as having it in crash carts and emergency kits in the operating room or anesthesia workstations. Additionally, simulation-based training should be practiced to assess and improve the readiness of the medical staff, ultimately improving patient outcomes.
